# The impact of socioeconomic factors on the efficiency of voluntary toxoplasmosis screening during pregnancy: a population-based study

**DOI:** 10.1186/s12884-016-0966-0

**Published:** 2016-07-29

**Authors:** A. E. Lange, J. R. Thyrian, S. Wetzka, S. Flessa, W. Hoffmann, M. Zygmunt, C. Fusch, H. N. Lode, M. Heckmann

**Affiliations:** 1 Department of Pediatrics and Neonatology & Paediatric Intensive Care, University Medicine Greifswald, F.-Sauerbruchstr, 17475 Greifswald, Germany; 2 Division of Health Care Epidemiology and Community Health, Institute of Community Medicine, University of Greifswald, Greifswald, Germany; 3 Department of Health Care Management, Faculty of Law and Economics, Ernst-Moritz-Arndt-University of Greifswald, Greifswald, Germany; 4 Department of Gynaecology and Obstetrics, University of Greifswald, Greifswald, Germany; 5 Division of Neonatology, Department of Pediatrics, McMaster University, Hamilton, ON Canada

**Keywords:** Toxoplasmosis, Screening, Public health, SNiP-Study, Screening pregnancy, Toxoplasmosis infection, Toxoplasmosis screening, Private healthcare services

## Abstract

**Background:**

Congenital toxoplasmosis is associated with severe complications. German state health insurance covers rubella, but not toxoplasmosis, immunity screening. We analysed the effect of socioeconomic factors on the efficiency of private toxoplasmosis screening during pregnancy.

**Methods:**

Toxoplasmosis and rubella screening data (*n* = 5402 mothers) were collected within the population-based Survey of Neonates in Pomerania (SNiP).

**Results:**

At the first-trimester screening, 34.4 % (88.1 %) of expecting mothers were immune to toxoplasmosis (rubella). Susceptibility for toxoplasmosis (rubella) was observed in 39.6 % (8.9 %) and 25.8 % (2.95 %) were not tested. Data on a 2^nd^ screening were available in a subgroup of women with negative immunity showing less than 45 % participation rate. Active toxoplasmosis (no rubella) infection was observed in 0.3 % (*n* = 17) of pregnant women. A multiple logistic regression model (AIC = 719.67; AUC = 0.725) revealed that the likelihood of participating in a second toxoplasmosis screening increased among women with a good level of education and a steady partnership and decreased with paternal unemployment and the absence of breastfeeding. The highest probability of non-participation in toxoplasmosis screening was found among women with temporal burden and family responsibilities. A cost-benefit analysis showed that covering general screening for toxoplasmosis with health insurance saved costs.

**Conclusion:**

Toxoplasmosis carried a substantial risk of infection during pregnancy. Although increased socioeconomic status was positively associated with the participation in toxoplasmosis screening, this was not the case when pregnant women had strong temporal burden and family responsibilities. This data supports the need for toxoplasmosis screening among pregnant women as a general healthcare benefit covered by insurance.

**Electronic supplementary material:**

The online version of this article (doi:10.1186/s12884-016-0966-0) contains supplementary material, which is available to authorized users.

## Background

The protozoan *Toxoplasma gondii* is a major challenge to public health. The overall rate of toxoplasmosis infection during pregnancy varies from 1 to 120 per 10,000 births, depending on environmental conditions [1, 2] Although the German Ministry of Health reported eight cases of acute gestational toxoplasmosis infection in 2009, higher rates seem likely [3].

Following maternal seroconversion, the estimated average risk of fetal infection during pregnancy is 40–50 %. Fetal risk correlates inversely with the diaplacental acquisition rate, which increases from approximately 2 % during the first trimester to 30 % during the second trimester, peaking at approximately 80 % immediately before delivery [1, 4–6].

During the first two trimesters, acute toxoplasmosis infection is characterised by septic symptoms, hepatosplenomegaly, thrombocytopenia, hyperbilirubinemia, and central nervous system infections [1, 7, 8] The latter typically present with encephalitis in combination with retinochorioiditis, hydrocephalus, intracranial calcifications, microphthalmia, and microcephaly, as well as calcifying necroses developing from reactive inflammations, to the point of spontaneous abortion [9]. In contrast, the majority of fetuses infected during the third trimester lack pathological findings at birth (70–90 %) [10, 11]. However, in 30–70 % of offspring with clinical abnormalities, those abnormalities are not detected initially; these children typically have chorioretinitis, hearing loss, and mental retardation later in life [12, 13]. *T. gondii* infections are mostly asymptomatic in adults and immunocompetent individuals; consequently, acute infections during pregnancy usually go unnoticed [8, 14].

Effective prevention strategies are crucial. One possibility is to provide prophylactic therapy to seroconverted women during pregnancy [15]. When infection is suspected, materno-fetal therapy may be initiated at an early stage. Depending on the gestational week, standardised therapy regimes of different durations markedly alleviate the typical course of toxoplasmosis in neonates [16, 17]. Knowledge of negative immune status would also enable women to take appropriate preventive precautions [18]. In cases of negative immunity, screenings are conducted at 3-month intervals during gestation to detect possible infections [16, 19].

In Germany, resident gynaecologists offer toxoplasmosis screening and the cost of toxoplasmosis screening is borne by the individual. In contrast, rubella screening is covered by state health insurance, although an anti-rubella vaccine is available. The prevalence of rubella vaccination among German children approaches 75 %, depending on where they reside. Similar to toxoplasmosis, the transmission rate of rubella during pregnancy also depends on the time of maternal infection. Sufficient anti-rubella immunity excludes congenital rubella syndrome throughout pregnancy. Women without sufficient immunity are re-tested later in pregnancy. At the end of pregnancy, a booster vaccine is recommended for mothers with negative immune status [20].

The risk of intrauterine toxoplasmosis infection is higher than that of rubella infection. Less severe disease is commonly reported in countries in which prenatal screening and treatment have been systematically implemented (e.g., France). Gravidic seroconversions (and therefore cases of congenital toxoplasmosis) were reduced in France after toxoplasmosis screening was implemented [21].

Regarding efficacy, there is always the question of to what extent health and monetary concerns can be weighed against each another. A cost-benefit analysis intended to assess the efficacy of a screening program should compare the total cost (the cost of screening and the cost of treatment in cases of seroconversion) with the cost of treatment, rehabilitation, and (in the worst cases) lifelong disability before and after the reduction of cases. Ideally, the screening cost should be equal to or less than the cost of moderating congenital toxoplasmosis.

We used population-based data from the Survey of Neonates in Pomerania (SNiP) to analyse the extent to which toxoplasmosis screening as a privately paid service is used compared with rubella screening (a standard, insurance-paid service), and whether toxoplasmosis screening utilisation correlates with socioeconomic factors.

## Methods

### Study design

The present study is part of the population-based birth cohort study “Survey of Neonates in Pomerania (SNiP)”, conducted from 2002 to 2008. Physicians specially trained for the study collected data about pregnancy and births at the participating hospitals. Detailed information about newborn children and their mothers regarding neonatal health, morbidity, and mortality was collected to calculate prevalence rates for major neonatal diseases, risk factors, and confounding conditions on a cross-sectional and prospective basis. According to census data, 7220 babies were born in the study region of Pomerania in northeast Germany during the study period. In SNiP, data from *n* = 6828 (95 %) babies and their respective mothers (*n* = 6747) were assessed, yielding high population coverage. Exclusions and non-responders comprised *n* = 1556 individuals; a minimum dataset was compiled comprising data on the health status of these newborns and women, but lacking detailed information about environmental parameters. From the mothers, personal data, medical records (149 variables), a personal interview (84 variables), and a self-administered questionnaire concerning socioeconomic background (40 variables) were recorded. Details of the SNiP study have been reported by Ebner et al. [22].

### Population

Data from 5402 mothers of neonates delivered between May 2002 and June 2008 were analysed for toxoplasmosis and rubella screening, infection, and socioeconomic background.

### Data assessment

All mothers provided written informed consent to participate in the study, which was approved by the Ethics Committee of the Ernst Moritz Arndt University, Greifswald. Data was collected in standardised 5- to 10-min interviews. Parents were also asked to complete a questionnaire during their stay on the ward and return it to the medical staff before discharge. This questionnaire included questions about the parents’ social background and lifestyle. Data about the gestational period and any preventive examinations was acquired using the mother’s medical file and maternity card. The collected data was anonymised and stored in an Access database.

Toxoplasmosis and rubella screening data from 5402 mothers was evaluated. This data contained information about immunity against toxoplasmosis and rubella (yes/no/infection during pregnancy/no information available). Variables were correlated with family status, education level, gainful employment, and income.

To study toxoplasmosis screening attendance as a preventive measure, women were classified into two groups according to whether or not they underwent toxoplasmosis screening. Women who underwent toxoplasmosis screening were stratified into three categories: with positive immunity, defined as proof of IgG antibodies in maternal serum, proof of IgG antibodies in umbilical cord blood, and no evidence of IgM antibodies in umbilical cord blood (Category I); with negative immunity, defined as no proof of IgG antibodies in maternal serum, no proof of IgM antibodies, and no evidence of IgG or IgM antibodies in umbilical cord blood (Category II); and with toxoplasmosis infection during pregnancy, defined as proof of IgM and IgG antibodies in maternal blood or proof of IgM and IgG antibodies in umbilical cord blood (Category III).

For laboratory analysis the Serion Elisa classic Toxoplasmosis gondii IgG/IgM tests (Vision/Serion GmbH, Würzburg, Germany) were used. (Vtox IgG <10UIE) and (vtox IgM <300 UIE) demonstrate a negative result. Applying these threshold values result in a sentivity of 98.2 % (97.8 %) and a specificity of 99.4 % (95.7 %) for IgG (IgM) (information supplied by the manufacturer). Placental leak occasionally can lead to false-positive IgM reaction. In these cases, the infants were tested again several days later, because the half-life of IgM is only approximately 5 days. A persistent positive or increasing IgG titer in the infant and/or a positive IgM indicated congenital infection. Toxoplasmosis PCR in amniotic fluid was not routinely applied.

In conjunction we use the IgG Avidity Assay. The method is most useful in woman in the first 16 weeks of gestation in whom IgM antibodies are found. It also is useful late in gestation to determine whether infection was acquired 4 or more months earlier, thereby allowing for an estimate of the rate of fetal infection at a given time during gestation [1].

### Statistical analysis

All data were stored using a Microsoft Access 2002 (Microsoft Corporation, Redmond, WA, USA) database. Statistical calculations were performed using SPSS (IBM SPSS 21.0, IBM Deutschland GmbH, Munich, Germany) for Windows 7® (Microsoft Corporation) and R (R Development Core Team, 2011). The mean (SD), median (range), or numbers of patients were processed for each baseline characteristic and are depicted separately for socioeconomic factors and screening utilisation. Simple logistic regression, with screening outcome as a dependent variable and socioeconomic factors as independent variables, was used to select variables that were associated significantly with screening rate. Subsequently, we evaluated these associations (all variables with *P* < 0.1) in a multiple logistic regression model (OR 97.5 % CI), taking into account confounders such as the 40 socioeconomic variables mentioned in the Study Design subsection. *P*-values <0.05 were considered statistically significant.

### Health economic evaluation of general screening

We developed a simple decision tree (Additional file 1) to estimate whether financial coverage of general toxoplasmosis screening by health insurance would be beneficial. We compared the expected value of total direct and indirect costs for two scenarios (represented in the two primary branches of the decision tree). Scenario 1 represented complete coverage of screening costs by health insurance, resulting in higher screening participation and a correspondingly lower number of cases. Scenario 2 represented the status quo in Germany (screening paid for out-of-pocket). The main input variables of the model were the costs of screening, treatment, and indirect costs (loss of lifelong productivity), as well as the likelihoods of screening (subject to scenarios 1 and 2), toxoplasmosis, treatment, and death. Data was obtained from literature [23] and adjusted using findings from SNiP.

## Results

### Study population

**Fig. 1:**
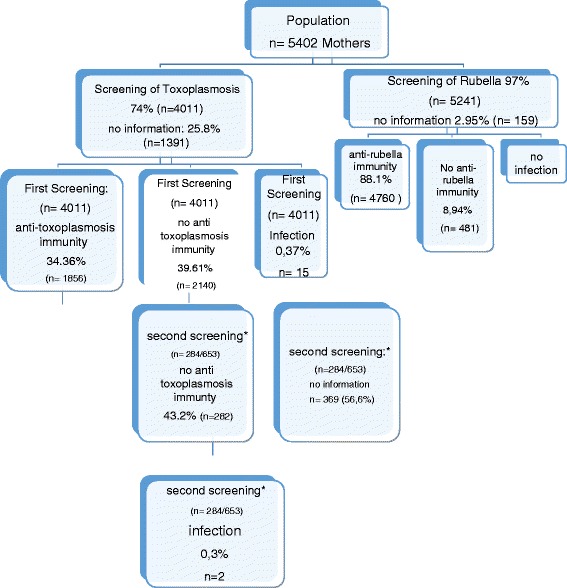
Participation of pregnant women in toxoplasmosis and rubella screening. Data on a 2nd screening were available in a subgroup of women from 2002–2003

For all pregnant women (*n* = 5402), utilisation rate for early rubella screening as a standard service was 97.05 %; the utilisation rate for toxoplasmosis screening as a private service was 74 % (Fig. 1).

### Screening results: toxoplasmosis vs. rubella immunity

#### First screening

At the first screening, the rate of anti-toxoplasmosis immunity (*n* = 1856; 34.36 %) was less than one-half the rate of anti-rubella immunity (*n* = 4760; 88.11 %). Screening revealed 2140 cases (39.61 %) that lacked toxoplasmosis immunity and 481 cases (8.94 %) that lacked rubella immunity. Data from the mothers’ medical files and maternity cards provided no information regarding toxoplasmosis immunity status for 1391 women (25.8 %) and regarding rubella immunity status for 159 women (2.95 %). Active toxoplasmosis infection during pregnancy was observed in 17 women (0.3 %, CI 95 0.2- 0.6). Infection occurred during pregnancy at gestational age of up to 10 weeks in *n* = 6 (35.3 %) cases, in week 11–18 in *n* = 6 (35.3 %), in 3 cases there were no information (Table 1). The mean gestational age of their newborns was 40.3 weeks (SD = 1.01). There was suspicious sonography of the skull in *n* = 2 (11.76 %) newborns and no hearing deficit in any of the newborns (neither right or left ear). Active infection was detected in cord blood from eight newborns (Table 1).

**Table 1:** Treatment and neonatal short-term outcome of 17 Pregnancies with maternal toxoplasma infection. SGA small for gestational age

Newborn	GA AT infection (weeks)	Mother IGM	Mother IGG	Avidity	Treatment spiramycin	Newborn IGG	Newborn IGM	Findings in the fetus	Treatment newborn
1	6	+	+	no	+	-	-	no	no
2	8	+	+	no	+	-	-	no	no
3	8	+	+	low	+	+	-	SGA	yes
4	8	+	+	low	+	+	+^a^	no	yes
5	10	+	+	low	?	+	-	no	no
6	10	+	+	low	-	+ IgG increased^a^	-	Cerebral ventricular dilatation	yes
7	11	+	+	low	+	+	-	no	no
8	12	+	+	low	inadequate	-	-	SGA	no
9	12	+	+	low	+	+	+^a^	SGA	yes
10	14	+	+	high	+	+ -^a^	-	no	no
11	16	+	+	low	-	+	+	Cerebral Disorders	yes
12	17	+	+	low	+	+	-	no	no
13	No informatio	+	+	low	+	+	-	no	no
14	No information	+	+	low	No information	+	-	no	no
15	No information	+	+	low	No information	+	-	no	no
16^b^	20	+	+	no	-	+	+	SGA	yes
17^b^	21	+	+	no	-	+	+	SGA	yes

^a^at repeated blood sampling, ^b^Newborn 16 and 17: detected at the second screening

No active rubella infections were documented.

#### Second screening

Six hundred fifty-three women who initially screened negative for toxoplasmosis were offered a second screening from May 2002 to March 2003; 284 (43.5 % of *n* = 653) declined a second screening (Fig. 1). Two hundred eighty-two women (43.2 %) had a negative screening result; two (0.31 %) had seroconverted since the first screening, indicating infection during early pregnancy.

### Impact of socioeconomic factors on screening participation: univariate analysis

Details of the univariate analysis are available online as Supplementary Information.

#### Correlation of family status with first screening participation

Participation in the first toxoplasmosis screening correlated significantly with the family status of pregnant women (*n* = 3999, *p* > 0.001). Married pregnant women living with a spouse had the highest participation rate (74.5 %); married women separated from a spouse had a 60 % participation rate (Additional file 2: Table S1). Single pregnant women participated in the first toxoplasmosis screening at a much higher rate (75.5 %) than married pregnant women not living with a spouse (60.0 %) or divorced pregnant women (59.8 %).

In contrast, we observed no statistically significant correlation between participation in rubella screening and family status (*n* = 3999 [62.91 % of the 5402 women included in analysis], *p* = 0.42). Married women and married women living separated from a spouse took part in rubella screening at high rates (97.3 and 91.1 %, respectively). Rubella screening participation was also high among single (97.9 %), divorced (98.3 %), and widowed (100 %) women.

#### Correlation of patient-reported education with first screening participation

Education level and participation in toxoplasmosis and rubella screenings correlated positively (*n* = 4813 [89.1 % of the 5402 women included in analysis], *p* < 0.001). Mothers who had 8 years of secondary school and were university-qualified (Abitur, 83.4 %), had 8 years of secondary school (Fachhochschulreife, similar to A-levels, 78.50 %), and had 6 years of secondary school (Realschulabschluss, 76 %) participated more often in toxoplasmosis screening than mothers who had 5 years of secondary school (Hauptschulabschluss, 61.1 %), did not earn a school diploma (47.6 %), or were still in school (44.4 %). Participation in rubella screening was high across all educational levels (Additional file 3: Table S2).

#### Correlation between income level and first screening participation

Expectant mothers’ income level correlated positively with participation in the first toxoplasmosis screening (*n* = 2938 [54.38 % of the 5403 women included in the analysis], *p* < 0.001) (Additional file 4: Table S3). This correlation was not observed for rubella screening.

#### Influence of socioeconomic factors on participation in the second toxoplasmosis screening

Attendance at the second toxoplasmosis screening was higher among women with a higher education level and women who had been gainfully employed before pregnancy (Additional file 5: Table S4).

### Impact of socioeconomic factors on first screening participation: multiple logistic regression analysis

We calculated a multiple logistic regression model to evaluate the associations (all variables with *p* < 0.1) between screening participation and each of the studied socioeconomic factors (family status, education level, income level, steady partnership, gainful employment, planned pregnancy, and breastfeeding) (OR 97.5 % CI; Table 2). The model demonstrated a modest fit (AIC = 719.67; AUC = 0.725). When socioeconomic factors were viewed as cofounders, the likelihood of participating in a second toxoplasmosis screening increased among women with a good level of maternal education and a steady partnership. The likelihood of non-participation in an adequate toxoplasmosis screening increases by 1.14 with paternal unemployment and by 1.72 in the absence of breastfeeding.

**Table 2:** Impact of socioeconomic factors on first screening participation: multiple logistic regression analysis with non-participation in toxoplasmosis screening as the dependent variable

	OR	CI 2.50 %	97.5 % Sign test *p*-value	*p*
Solid partnership (=yes)	0.39	0.18	0.78	0.0115*
Maternal education level (=high)	0.68	0.57	0.80	0.000***
Job situation, mother (=employed)	1.14	1.03	1.25	0.0095**
Job situation, father (=employed)	0.86	0.69	1.14	0.0487*
Planned pregnancy	1.4	1.10	1.79	0.0071**
Absence of breastfeeding	1.7	1.35	2.18	0.000***

^a^ *n* = 3399 women (62.91 % of the 5402 included in the analysis)

***p < 0.001; **p < 0.01; *p < 0.05

Table 3 shows the probability of non-participation in toxoplasmosis screening when socioeconomic variables were combined in different ways. The highest probability of non-participation (66 %) was found in pregnant women with a high educational level, full-employed, planned their pregnancy, did not intend to breast-feed and lived in a steady partnership with an unemployed partner.

**Table 3:** Probability of non-participation in toxoplasmosis screening

Solid partnership	Education level, mother	Job situation, mother	Job situation, father	Planned pregnancy	Breastfeeding	Probability of non-participation
Yes^a^	Yes^b^	Full Time	No education	Yes	No	66.01 %
Yes	Yes	Full Time	No education	Yes	No	62.92 %
Yes	Yes	Unemployed	At school	Yes	No	61.97 %
Yes	Yes	Unemployed	At school	Yes	No	58.95 %
Yes	Yes	Unemployed	No education	Yes	Yes	55.42 %
Yes	Yes	Full Time	At school	No	No	54.76 %
Yes	Yes	Full Time	At school	No	No	54.76 %

^a^ *n* = 4813 women (89.1 % of the 5402 included in the analysis)

^b^mother in a stable partnership

^c^mother has completed a professional education

### Health economic evaluation of general screening

Based on the decision tree model (Fig. 1) and the data provided by [23], we estimated the expected lifelong cost as $633 USD for full coverage by health insurance and $1406 USD for out-of-pocket payment. From a societal perspective, coverage of toxoplasmosis screening by health insurance is advisable.

## Discussion

Substantial pathology in newborns, insufficient screening strategies, and the lack of a vaccine contribute to *T. gondii* as a considerable public health concern. In our cohort, sufficient immunity was detected in only 34.4 % (*n* = 1856) of mothers-to-be; 25.8 % (*n* = 1391) of women were never screened, and approximately 40 % (*n* = 2140) were initially diagnosed as lacking immunity without sufficient follow-up. The latter population urgently requires counselling regarding preventative measures and re-screening. In contrast, 88 % (*n* = 4760) of pregnant women were effectively protected against rubella. To avoid potential gestational toxoplasmosis infection, the immune status of pregnant women should be monitored at 3-month intervals [8]. But even worse, less than 45 % of women without immunity to toxoplasmosis participated in a second screening.

Our prevalence data indicate a gestational infection rate of 0.3 % (*n* = 17/5402), and active infection was detected in cord blood from eight newborns (8/3645; 0.22 %). These numbers are considerably higher than the recently published report that 8.5 in 10,000 pregnant women have toxoplasmosis, and 1.0 per 10 000 infants had congenital toxoplasmosis (13 % mean transmission rate) in Austria [24]. While in Austria toxoplasmosis screening in pregnancy is covered by national healthcare providers, this is not the case in Germany. The Law on the Prevention of infection provides anonymous mandatory reporting to the Robert-Koch-Institute (German Ministry of Health). But only 11 cases of active congenital toxoplasmosis were reported in 2008, [3] modelled calculations based on our study data yielded much higher rates, in agreement with other published calculations [8]. Furthermore, congenital toxoplasmosis often goes unreported; cases may not be detected until patients experience manifestations as adults [25].

A retrospective analysis at the Ludwig-Maximillian-University in Munich (LMU) reported on toxoplasmosis screening in 15,856 pregnancies between 2001 and 2008 [26, 27]. Toxoplasmosis serology was completed in 39.99 %; of these, 62.47 % were seronegative and 13.02 % were seropositive. Seroconversion was discovered in 0.14 % of these women, resulting in a calculated incidence of <0.057 %. This difference in immunity level may be explained by geography: LMU patient population is urban, while participants in the SNiP study live in Mecklenburg-Western Pomerania. In the latter state, which is dominated by farming communities, women are exposed to a greater environmental risk of contracting toxoplasmosis (seropositive: SNiP 34.36 % vs. LMU 13.02 %; seronegative: SNiP 39.61 % vs. LMU 62.47 %).

Each screening is subject to a fee that is paid by the individual [25]. Therefor, the current screening strategy (screening as a private service) may be one reason for the insufficient number of toxoplasmosis screenings. Although one screening during pregnancy is sufficient for women with positive immune status, cases that lack immunity require three examinations to identify gestational infections in adequate time to initiate materno-fetal therapy [25]. We contrast this situation against rubella screening, a standard service covered by state health insurance. The rate of maternal participation is 23 % higher for rubella screening than for toxoplasmosis screening, although vaccination renders the rate of rubella immunity (88.11 %) much higher than that of toxoplasmosis immunity (34.36 %).

Socioeconomic factors also have a marked negative influence on the rate of screening participation. We have demonstrated that attendance at toxoplasmosis screenings is higher among mothers with higher education levels and gainful employment preceding pregnancy.

In this study, lower socioeconomic status was a clear risk factor for non-participation in toxoplasmosis screening. Toxoplasmosis is more likely to occur among neonates born to mothers in the lower social strata. Although increased socioeconomic status was positively associated with the participation in toxoplasmosis screening, this was not the case when pregnant women had strong temporal burden and family responsibilities. Our model demonstrated that mothers who are in a long-term relationship, have graduated from school, are working full-time, with a partner who is not working full-time (student, in training), undergoing an intended/planned pregnancy, and with no intention of breastfeeding are least likely to make use of toxoplasmosis screening programs (Table 3). These effects may not be observed as strongly in rubella screenings as the costs for this are covered by public insurance, whereas toxoplasmosis screenings are not and thus also require additional consulting with the responsible physicians. This is associated with more time having to be contributed by the mother, which may have aggravated the negative effect on participation rate in toxoplasmosis screening.

Our analysis shows that the expected lifelong cost for out-of-pocket payment are 2.2 fold the cost for full coverage by health insurance, i.e., the society could save 773 USD per pregnancy if the health insurance had the obligation to pay for screening for toxoplasmosis. This analysis is preliminary as some potential arms of the decision tree (Fig. 1) are not included, such as false positive sero-conversions with unnecessary amniocentesis and fetal loss. However, with a ratio of 2.2 the results are quite robust and allow the general statement that screening is cost-effective. This finding is in line with prior findings that general toxoplasmosis screening is highly cost-efficient from a societal perspective [23], but more research on the economics of toxoplasmosis screening is needed to address the complete complexity of the epidemiological, economic and social situation.

Stillwaggon et al. [23] we highlight a general dilemma of healthcare financing: cost-effectiveness of general screening from a societal perspective does not ensure implementation by health insurance. The high figures provided by Stillwaggon et al [23] include indirect costs (i.e., lifelong loss of productivity, fatalities), which are irrelevant from the perspective of health insurance. The asymmetry of societal and institutional benefits regularly requires legal action (e.g., the obligatory financing of toxoplasmosis screening by health insurance). Our findings also warrant additional research regarding the health economics of so-called neglected diseases (Fig. 1).

Because the risk of toxoplasmosis infection is higher in mothers-to-be with negative immune status, they should be screened three times during pregnancy to detect potential seroconversion as early as possible. In our study we found 15 cases of toxoplasmosis out of 4011 woman at the first screening, which means that 267 mothers were screened to detect one case of toxoplasmosis. Furthermore, the rate of mothers with maternal toxoplasmosis who were treated to prevent one treatment in the offspring was 9/3 (Table 1) [28]. In France, this screening strategy reduced congenital toxoplasmosis cases by 10-fold [21]. Furthermore, the Austrian toxoplasmosis register reported recently that early detection of toxoplasmosis in pregnancy allowed adequate treatment with a 6.fold reduction in transmission rate [24].

In our cohort, participation has to be regarded as inadequate during toxoplasmosis screenings at all stages.

A limitation of our study is the lack of serologic follow up data of newborns at risk during the first year of life. A second limitation is that data on the acceptance of a 2^nd^ screening derived only from a subgroup of our cohort. These limitations may have resulted in a higher incidence of both toxoplasmosis infections in pregnancy and in the newborn in our population.

In conclusion, private toxoplasmosis screening in pregnancy carries a substantial risk of undiagnosed infection. Participation in toxoplasmosis screening was influenced by socioeconomic factors. Increased socioeconomic status was positively associated with the participation in toxoplasmosis screening. But this was not the case when pregnant women had strong temporal burden and family responsibilities. It seems reasonable to make toxoplasmosis screening a standard of care and a reimbursable healthcare benefit for all pregnant women, as our data show that large numbers of pregnant women do not participate adequately in this screening. These considerations reflect the urgent need for a globally applicable and consistent toxoplasmosis screening strategy for pregnant women. Such a strategy could improve awareness, knowledge, and prevention, and could guarantee early treatment in cases of gestational infection. Taking into account the potential follow-up costs for neonates with undiagnosed toxoplasmosis infections, health economic considerations may also support the general screening of all women while trying to conceive and/or during early pregnancy.

## Conclusion

Toxoplasmosis carried a substantial risk of infection during pregnancy. Although increased socioeconomic status was positively associated with the participation in toxoplasmosis screening, this was not the case when pregnant women had strong temporal burden and family responsibilities. This data supports the need for toxoplasmosis screening among pregnant women as a general healthcare benefit covered by insurance.

Taking into account the potential follow-up costs for neonates with undiagnosed toxoplasmosis infections, health economic considerations may also support the general screening of all women while trying to conceive and/or during early pregnancy.

## Abbreviations

AIC, Area in the curve; AUC, Area under the curve; CI, Confidence interval; e.g, for example; IgG, Immunoglobulin G; IgM, Immunglobuline M; LMU, Ludwig Maxilmilian Universtiy Munich; OR, Odds ratio; PCR, polymerase change reaction; SD, standard deviation; SNIP, Survey of Neonates in Pomerania; T.gondii, Toxoplasma gondii; UIE, unit international; USD, United States Dollar.

## Additional files

Additional file 1: A Simple Decision Tree Model of Health Economic Evaluation of General Toxoplasmosis Screening. (TIF 612 kb) Additional file 2: Table S1. Correlations between family status and participation in first toxoplasmosis and rubella screenings ^a^ *n* = 4813 women (89.1 % of the 5402 included in the analysis). ***p* < 0.001; **p* = 0.42 regarding participation vs. lack of participation. All data are presented as percentages. (DOCX 11 kb) Additional file 3: Table S2. Participation in first toxoplasmosis and rubella screenings with respect to education level, *n* = 4813 (89.1 % of 5402) women included in the analysis. All data are presented as percentages.**p* < 0.05; ***p* < 0.01; ****p* < 0.001; *****p* < 0.0001. (DOCX 12 kb) Additional file 4: Table S3. Participation in first toxoplasmosis or rubella screening with respect to income level, *n* = 4813 (89.1 % of 5402) women included in the analysis. All data are presented as percentages.**p* < 0.05; ***p* < 0.01; ****p* < 0.001; *****p* < 0.0001. (DOCX 11 kb) Additional file 5: Table S4. Influence of socioeconomic factors on participation in second toxoplasmosis screening, *n* = 4813 (89.1 % of 5402) women included in the analysis. All data are presented as percentages. **p* < 0.05; ***p* < 0.01; ****p* < 0.001; *****p* < 0.001. (DOCX 12 kb)

## References

[1] Remington & Klein, Wilson & Baker, Infectious Diseases of the Fetus and Newborn Infant, 2006 sixth Edition:31:948- 1091

[2] Tenter A, Heckeroth AR, Weiss LM (2000). Toxoplasma gondii: from animals to humans. Int J Parasitol.

[3] Robert-Koch-Institut. Infectionsepidemiologisches Jahrbuch 2008.

[4] Berrebi A, Bardou M, Bessieres MH, Nowakowska D, Castagno R, Rolland M (2007). Outcome for children infected with congenital toxoplasmosis in the first trimester and with normal ultrasound findings: a study of 36 cases. Eur J Obstet Gynecol Reprod Biol.

[5] Hill D, Dubey JP (2002). Toxoplasma gondii: transmission, diagnosis and prevention. Clin Microbiol Infect.

[6] Robert-Gangneux F, Gavinet MF, Ancelle T, Raymond J, Tourte-Schaefer C, Dupouy-Camet J (1999). Value of prenatal diagnosis and early postnatal diagnosis of congenital toxoplasmosis: retrospective study of 110 cases. J Clin Microbiol.

[7] Hedriana HL, Mitchell JL, Brown GM, Williams SB (1993). Normal fetal outcome in a pregnancy with central nervous system toxoplasmosis and human immunodeficiency virus infection. A case report. J Reprod Med.

[8] Cristoph J, Kattner E, Seitz HM, Reiter-Owona I (2004). Strategien zur Diagnostik und Behandlung der pränatalen Toxoplasma-Infektion - ein aktueller Überblick. Z Geburtshilfe Neonatologie.

[9] Montoya JG, Liesenfeld O (2004). Toxoplasmosis. Lancet.

[10] Lebech M, Joynson DH, Seitz HM, Thulliez P, Gilbert RE, Dutton GN (1996). Classification system and case definitions of Toxoplasma gondii infection in immunocompetent pregnant women and their congenitally infected offspring. European Research Network on Congenital Toxoplasmosis. Eur J Clin Microbiol Infect Dis.

[11] Mombro M, Perathoner C, Leone A, Buttafuoco V, Zotti C, Lievre MA (2003). Congenital toxoplasmosis: assessment of risk to newborns in confirmed and uncertain maternal infection. Eur J Pediatr.

[12] Vutova K, Peicheva Z, Popova A, Markova V, Mincheva N, Todorov T (2002). Congenital toxoplasmosis: eye manifestations in infants and children. Ann Trop Paediatr.

[13] Safadi MA, Berezin EN, Farhat CK, Carvalho ES (2003). Clinical presentation and follow up of children with congenital toxoplasmosis in Brazil. Braz J Infect Dis.

[14] Dunn D, Wallon M, Peyron F, Petersen E, Peckham C, Gilbert R (1999). Mother-to-child transmission of toxoplasmosis: risk estimates for clinical counselling. Lancet.

[15] Pfaff AW, Candolfi E (2008). New insights in toxoplasmosis immunology during pregnancy. Perspective for vaccine prevention. Parassitologia.

[16] Roberts F, Roberts CW, Ferguson DJ, McLeod R (2000). Inhibition of nitric oxide production exacerbates chronic ocular toxoplasmosis. Parasite Immunol.

[17] Robert-Koch-Instiut (2009). Infektionsepidemiologisches Jahrbuch,Toxoplasmose 2009 Global Public Health.

[18] Elsheikha HM (2008). Safer food for pregnant women: Practices and risks. Public Health.

[19] Dollfus H, Dureau P, Hennequin C, Uteza Y, Bron A, Dufier JL (1998). Congenital toxoplasma chorioretinitis transmitted by preconceptionally immune women. Br J Ophthalmol.

[20] Best JM (2007). Rubella. Semin Fetal Neonatal Med.

[21] Ambroise-Thomas P, Schweitzer M, Pinon JM, Thiebaugeorges O (2001). Prevention of congenital toxoplasmosis in France. Risk assessment. Results and perspectives of prenatal screening and newborn follow up. Bull Acad Natl Med.

[22] Ebner A, Thyrian JR, Lange A, Lingnau ML, Scheler-Hofmann M, Rosskopf D, et al. Survey of Neonates in Pomerania (SNiP): a population-based birth study--objectives, design and population coverage. Paediatr Perinat Epidemiol. 24(2):190-9. Epub 2010/04/27. eng. 10.1111/j.1365-3016.2009.01078.x 20415776

[23] Stillwaggon E, Carrier CS, Sautter M, McLeod R (2011). Maternal serologic screening to prevent congenital toxoplasmosis: a decision-analytic economic model. PLoS Negl Trop Dis.

[24] Prusa AR, Kasper C, Pollak A, Gleiss A, Waldhoer T, Hayde The Austrian Toxoplasosis Register, 1992- 2008. Clin. Infect Dis.2014 Sep 12. Pii:ciu 724. [Epub ahead of print] 10.1093/cid/ciu724 25216688

[25] Gross U (2004). Prevalence and public-health-aspects of toxoplasmosis. Bundesgesundheitsblatt Gesundheitsforschung Gesundheitsschutz.

[26] Abboud P, Villena I, Chemla C, Leroux B, Talmuld M, Bednarczyk L (1997). Screening for congenital toxoplasmosis: pregnancy outcome after prenatal diagnosis in 211 cases. J Gynecol Obstet Biol Reprod.

[27] [Comment on the publication by Abholz, H. H.: Screening for toxoplasmosis in pregnancy: more risk than benefit]. Gesundheitswesen. 1994 Jul;56(7):411-2. Zur Publikation Abholz, H.H.: Toxoplasmose-Screening in der Schwangerschaft: mehr Schaden als Nutzen. 7919706

[28] Robert- Gangneux F (2014). It is not only the cat that did it: how to prevent and treat congenital toxoplasmosis. Infect.

