# Supplementary figures and images for: The impact of socioeconomic factors on the efficiency of voluntary toxoplasmosis screening during pregnancy: a population-based study
Source: BMC Pregnancy Childbirth. 2016 Jul 29;16:197. doi: 10.1186/s12884-016-0966-0 (PMC4966761; doi:10.1186/s12884-016-0966-0)

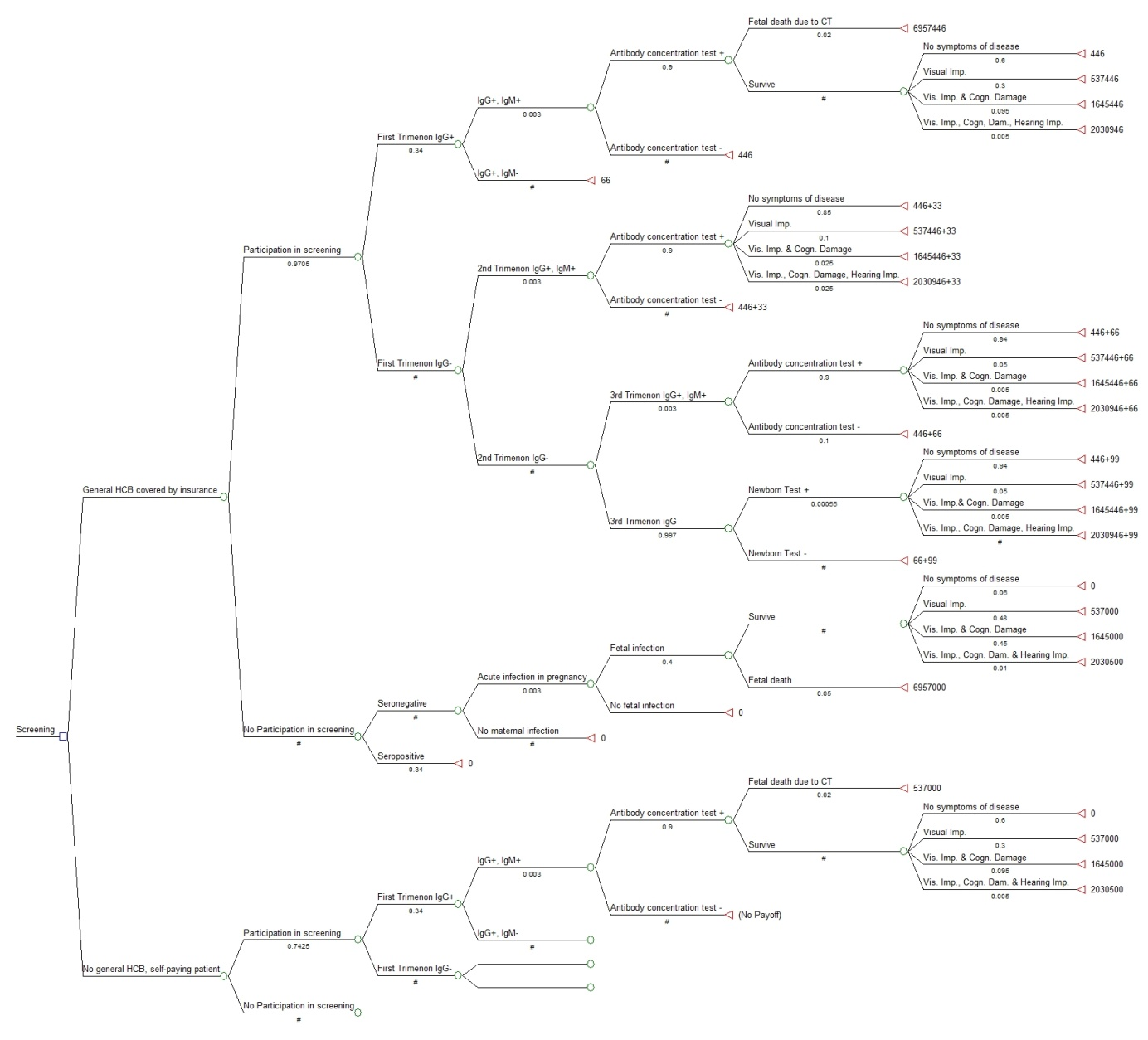

Supplement: Additional file 1: — A Simple Decision Tree Model of Health Economic Evaluation of General Toxoplasmosis Screening. (TIF 612 kb) [file 12884_2016_966_MOESM1_ESM.tif]
