# Supplementary material for: The impact of socioeconomic factors on the efficiency of voluntary toxoplasmosis screening during pregnancy: a population-based study
Source: BMC Pregnancy Childbirth. 2016 Jul 29;16:197. doi: 10.1186/s12884-016-0966-0 (PMC4966761; doi:10.1186/s12884-016-0966-0)
Supplement: Additional file 2: Table S1. — Correlations between family status and participation in first toxoplasmosis and rubella screenings a n = 4813 women (89.1 % of the 5402 included in the analysis). **p < 0.001; *p = 0.42 regarding participation vs. lack of participation. All data are presented as percentages. (DOCX 11 kb) [file 12884_2016_966_MOESM2_ESM.docx]

**Supplementary Table 1 Correlations between family status and participation in first toxoplasmosis and rubella screenings**

| **Family status ͣ** | **Participation in first toxoplasmosis screening, %**** | **Participation in first rubella screening, %*** |
| --- | --- | --- |
| Married | 74.5** | 97.3* |
| Married but separated | 60.0** | 91.1* |
| Single | 75.5** | 97.9* |
| Divorced | 59.8** | 98.3* |
| Widowed | 100 | 100 |

^a^n =4813 women (89.1% of the 5402 included in the analysis)

**p<0.001; *p=0.42 regarding participation vs. lack of participation

All data are presented as percentages.
