# Supplementary material for: The impact of socioeconomic factors on the efficiency of voluntary toxoplasmosis screening during pregnancy: a population-based study
Source: BMC Pregnancy Childbirth. 2016 Jul 29;16:197. doi: 10.1186/s12884-016-0966-0 (PMC4966761; doi:10.1186/s12884-016-0966-0)
Supplement: Additional file 3: Table S2. — Participation in first toxoplasmosis and rubella screenings with respect to education level, n = 4813 (89.1 % of 5402) women included in the analysis. All data are presented as percentages.*p < 0.05; **p < 0.01; ***p < 0.001; ****p < 0.0001. (DOCX 12 kb) [file 12884_2016_966_MOESM3_ESM.docx]

**Supplementary Table 2 Participation in first toxoplasmosis and rubella screenings with respect to education level**

| **N= 4813/5402 (89%)** | **Participation in first toxoplasmosis screening****** | | | | **Participation in first rubella screening** | | | |  |
| --- | --- | --- | --- | --- | --- | --- | --- | --- | --- |
| Secondary school, qualifying for university (Abitur) |  | 83.4*** |  |  |  | 85.5* |  |  | |
| Secondary school, qualifying for college (Fachhochschulreife) |  | 78.5*** |  |  |  | 84.7 |  |  | |
| Secondary school, advanced level (Realschule) |  | 76*** |  |  |  | 80.8 |  |  | |
| Secondary school, basic level (Hauptschule) |  | 61.1*** |  |  |  | 82.6 |  |  | |
| Still at school |  | 44.4 |  |  |  | 73.7 |  |  | |
| None |  | 48.8*** |  |  |  | 82.1 |  |  | |
| Other |  | 64.4** |  |  |  | 77.8 |  |  | |
| Not available |  | 65.8** |  |  |  | 86.6 |  |  | |

n=4813 (89.1% of 5402) women included in the analysis.

All data are presented as percentages.

*p<0.05; **p<0.01; ***p<0.001; ****p<0.0001
