# Supplementary material for: The impact of socioeconomic factors on the efficiency of voluntary toxoplasmosis screening during pregnancy: a population-based study
Source: BMC Pregnancy Childbirth. 2016 Jul 29;16:197. doi: 10.1186/s12884-016-0966-0 (PMC4966761; doi:10.1186/s12884-016-0966-0)
Supplement: Additional file 4: Table S3. — Participation in first toxoplasmosis or rubella screening with respect to income level, n = 4813 (89.1 % of 5402) women included in the analysis. All data are presented as percentages.*p < 0.05; **p < 0.01; ***p < 0.001; ****p < 0.0001. (DOCX 11 kb) [file 12884_2016_966_MOESM4_ESM.docx]

**Supplementary** **Table 3 Participation in first toxoplasmosis or rubella screening with respect to income level**

| **Income level, Euros (n=2938/5402)** | **Participation in first toxoplasmosis screening (p< 0.001)***** | **Participation in first rubella screening (p= 0.383)** |
| --- | --- | --- |
| <1250 (n=1094) | 68.5*** | 96.9 |
| 1250-3000 (n=1319) | 76.5*** | 97.6 |
| >3000 (n= 515) | 82.9** | 98 |
| No information | 26.4 | 65.8 |

n=4813 (89.1% of 5402) women included in the analysis

All data are presented as percentages.

*p<0.05; **p<0.01; ***p<0.001; ****p<0.0001.
