# Supplementary material for: The impact of socioeconomic factors on the efficiency of voluntary toxoplasmosis screening during pregnancy: a population-based study
Source: BMC Pregnancy Childbirth. 2016 Jul 29;16:197. doi: 10.1186/s12884-016-0966-0 (PMC4966761; doi:10.1186/s12884-016-0966-0)
Supplement: Additional file 5: Table S4. — Influence of socioeconomic factors on participation in second toxoplasmosis screening, n = 4813 (89.1 % of 5402) women included in the analysis. All data are presented as percentages. *p < 0.05; **p < 0.01; ***p < 0.001; ****p < 0.001. (DOCX 12 kb) [file 12884_2016_966_MOESM5_ESM.docx]

**Supplementary** **Table 4** Influence of socioeconomic factors on participation in second toxoplasmosis screening

|  |  |  | | Yes, % | |
| --- | --- | --- | --- | --- | --- |
|  | Total |  |  |  | 43.5 |
| Education level**** | None |  |  |  | 60.0* |
|  | Secondary school, basic level |  |  |  | 34.7**** |
|  | Secondary school, advanced level |  |  |  | 41.3*** |
|  | Secondary school, qualifying for college |  |  |  | 53.3** |
|  | Secondary school, qualifying for university |  |  |  | 47.5*** |
|  | Others |  |  |  | 46.7 |
|  | Still in school |  |  |  | 0.0 |
|  | Information not available |  |  |  | 47.2 |
| Gainful employment*** | Gainfully employed |  |  |  | 44.0** |
|  | Unemployed |  |  |  | 40.8** |
|  | Others |  |  |  | 42.4 |
|  | Information not available |  |  |  | 50.0 |

n=4813 (89.1% of 5402) women included in the analysis.

All data are presented as percentages.

*p<0.05; **p<0.01; ***p<0.001; ****p<0.001
